# Bamboo shoot fermented products alleviated DSS-induced ulcerative colitis in mice by effectively controlling inflammatory reactions and adjusting the gut microbiota and its metabolites

**DOI:** 10.3389/fnut.2025.1724148

**Published:** 2025-12-19

**Authors:** Xiaona Lu, Yalin Xu, Cheng Zhang, Huijuan Liu, Jiao Xie, Bing Tian

**Affiliations:** 1 The Key Laboratory of Environmental Pollution Monitoring and Disease Control, Ministry of Education, School of Public Health, Guizhou Medical University, Guiyang, Guizhou, China; 2 Guizhou Agricultural Ecology and Resource Protection Station, Agriculture and Rural Affairs Department of Guizhou Province, Guiyang, Guizhou, China

**Keywords:** ulcerative colitis, fermented bamboo shoot, inflammatory factors, gut microbiota, SCFAs

## Abstract

**Background:**

Bamboo shoots have been shown to have anti-inflammatory, antioxidant, improving digestion and regulating intestinal metabolic disorders. Edible fermented foods are subject to complex reactions during the fermentation process, leading to the creation of new bioactive compounds and enzymes, which can be beneficial for the relief of symptoms of ulcerative colitis. However, research on the use of fermented bamboo shoots for alleviating ulcerative colitis symptoms is currently limited.

**Methods:**

The effects of fermented bamboo shoots on DSS-induced UC model mice were investigated and basic physicochemical indicators, such as the levels of inflammatory factors, the composition of gut microbiota and SCFA were analysed to investigate the mechanism through which fermented bamboo shoots mitigate UC.

**Results:**

The results revealed that fermented bamboo shoots significantly reduced disease severity in DSS-treated mice, as evidenced by body weight recovery, a decreased disease activity index, increased colon length, and recovery from tissue damage. Fermented bamboo shoots also reduced the secretion of the proinflammatory cytokines TNF-α, IL-1β and IL-6 (*p* < 0.01). In addition, fermented bamboo shoots significantly increased the abundance of *Akkermansia*, *Anaerovorax* and *Bacteroides* in the intestines (*p* < 0.01), as well as the levels of SCFAs including acetic acid, propionic acid, and butyric acid in the caecum contents (*p* < 0.01).

**Conclusion:**

In summary, fermented bamboo shoots may play a role in restoring the composition of intestinal microbiota and the production of their metabolites, which suggests that fermented bamboo shoots may be a functional dietary supplement for alleviating UC.

## 1 Introduction

Ulcerative colitis (UC) is a prevalent chronic inflammatory disease of the colon, commonly characterized by symptoms such as weight loss, diarrhoea and bloody, purulent stool (1). Currently, UC has become an increasing global health concern due to its high prevalence, frequent recurrence and the significantly elevated risk of colorectal cancer among affected individuals (1, 2). UC has led to the development of numerous therapies, among which biologics are the most common, used to treat UC without surgical treatment. Although biologics can improve the symptoms of UC to some extent, their limitations and side effects cannot be ignored. These include the potential for systemic infection, hyperlipidaemia, gastrointestinal disease and central nervous system disorders, which means that approximately 15% of patients may still require surgery following drug treatment (1, 3). Therefore, there is an urgent need to develop dietary supplements that are high efficacy, natural and easy to take.

Plant-based foods are recognised worldwide as a complementary or alternative form of medicine for treating a wide range of diseases (4). Among them, bamboo shoots, for example, have been shown to have anti-inflammatory and antioxidant properties, as well as improving digestion and regulating intestinal metabolic disorders (5, 6). Bamboo shoots conform to the theory of food and medicine homology in that they contain a substance that integrates pharmacological and nutritional functions into one entity (7). In addition, bamboo shoot dietary fibre has the potential to be used as a dietary supplement or nutraceutical with applications in the prevention of UC, according to Wu et al. (8). Edible fermented foods are products made from raw materials of vegetable and fruit materials that have been fermented by microorganisms (9, 10). During fermentation, particularly lactic acid fermentation, the various components of the raw material are broken down and synthesised by microorganisms via complex reactions. These reactions result in the formation of new active compounds, such as polyphenols, flavonoids, amino acids and organic acids, as well as enzymes, such as superoxide dismutase, lipases, proteases and amylases. These compounds enable the raw material to retain or even improve its nutritional value, while also increasing the utilisation rate of its biologically active compounds and enzymes by the human body. This can be beneficial for alleviating the symptoms of UC (11–14). Currently, there have been few studies conducted on the use of fermented bamboo shoots to relieve symptoms of UC, and further research is needed.

The pathogenesis of UC is often multifactorial and is generally considered a challenging disease. It is characterized by abnormal intestinal barrier function and gut microbiota dysbiosis, which are caused by genetic, socioenvironmental and psychological factors (2). Therefore, therapeutic strategies for UC focus on reducing inflammatory manifestations and restoring intestinal homeostasis. The gut microbiota has attracted much attention due to its key role in intestinal tissue formation, immune system development and nutrient uptake from food (15). Existing study has confirmed that the composition of the gut microbiota is closely linked to the onset and development of UC (16). For example, increased levels of intestinal pathogenic bacteria can stimulate the production of various cytokines, including tumour necrosis factor α (TNF-α) and interleukins (ILs). These cytokines can play a proinflammatory role in the intestinal tract, acting as mediators of intestinal mucosal damage and exacerbating the symptoms of UC (17, 18). In addition, beneficial gut microorganisms such as *Bifidobacterium*, *Lactobacillus* and *Prevotella*, can produce short-chain fatty acids (SCFAs) directly or indirectly. These include acetic acid, propionic acid and butyric acid (19). These SCFAs can alleviate the disrupted state of the gut microbiota in patients with UC by decreasing the abundance of pathogenic genera in the intestinal tract. They can also protect the morphology of intestinal epithelial cells and the integrity of the intestinal mucosa. At the same time, SCFAs can alleviate inflammation in the colon and accelerate cell proliferation. This strengthens the immune barrier of the intestinal tract and suppresses the immune-inflammatory response (20). Significant reductions in total SCFA, acetate, propionate and butyrate concentrations have been observed in patients with UC, compared with those in healthy individuals (21). In summary, SCFAs are key signalling molecules that mediate the interactions between diet, gut microbiota and host. Their production can be altered by dietary intake, which in turn regulates gut homeostasis, controls the production and release of inflammatory factors. This can slow or eliminate symptoms in patients with UC.

This study investigated the effects of fermented bamboo shoots on a dextran sulfate sodium (DSS)-induced UC model in mice. Basic physicochemical indicators, such as the levels of inflammatory factors and SCFAs, and the composition of gut microbiota were analysed to investigate the mechanism by which fermented bamboo shoots mitigate UC. The aim was to provide a theoretical basis for research into the application of fermented foods, as well as to provide a reference for the adjuvant treatment of patients with UC.

## 2 Materials and methods

### 2.1 Materials and reagents

Bamboo shoots (*Chimonobambusa utilis*) were obtained from Guifengte Food Co., Ltd. (Guizhou, China). C57BL/6J mice (SPF grade, male) were purchased from Beijing Huafu Kang Biotechnology Co., Ltd. [Beijing, China; Licence No. SCXK (Beijing) 2019-0008]. *Lactobacillus plantarum* and *Lactobacillus acidophilus* were purchased from Shandong Zhongke Jiayi Biological Co., Ltd. (Shangdong, China). Pectase and cellulose were purchased from Cangzhou Xiasheng Enzyme Biotechnology Co., Ltd. (Jiangsu, China). White granulated sugar was purchased from Yunnan Dianpeng Sugar Industry Co., Ltd. (Yunnan, China). DSS (MW 36,000–50,000 Da) was acquired from MP Biomedical (CA, United States). Salazosulfapyridine was sourced from Shanghai Xinyi Co., Ltd. (Shanghai, China). Paraformaldehyde (4%) was purchased from Beijing Ranjke Technology Co., Ltd. (Beijing, China). ELISA kits for IL-1β, TNF-α, and IL-6 were purchased from Novus Biologicals (Colorado, United States). MRS broth and HE staining kits were purchased from Solarbio (Beijing, China). The PAS staining kit was obtained from Beijing Reagan Biotechnology Co., Ltd. (Beijing, China).

### 2.2 Preparation of fermented bamboo shoots

The bamboo shoots were washed and dried and then homogenized with pure water at a material-liquid ratio of 1:10. Subsequently, enzymatic hydrolysis was performed on the homogenized liquid at an enzymatic rate of up to 71.68%, under the following conditions: a reaction time of 4 h, an enzyme addition of 1.0% (with a cellulase to pectinase ratio of 2:1), and a reaction temperature of 50 °C. After that, 6.5% lactic acid bacteria (*Lactobacillus plantarum* and *Lactobacillus acidophilus* at a 1:1 ratio) were added to the enzyme-hydrolysate, mixed in 8.1 g of sugar, and then fermentation was carried out at 38.5 °C for 49.0 h. Finally, vacuum freeze-drying was undergone by the samples to obtain fermented bamboo shoots. The final bamboo shoot fermentation products exhibited a viable bacterial count of 3.44 × 10^8^ CFU/mL and SOD activity of 24.12 U/mL. The non-fermented and fermented bamboo shoot components were analysed using ultra-high-performance liquid chromatography tandem mass spectrometry (UPLC-MS/MS) via a broad targeting technique, as shown in Supplementary Figure S1.

### 2.3 Animal experiments

**Figure 1:**
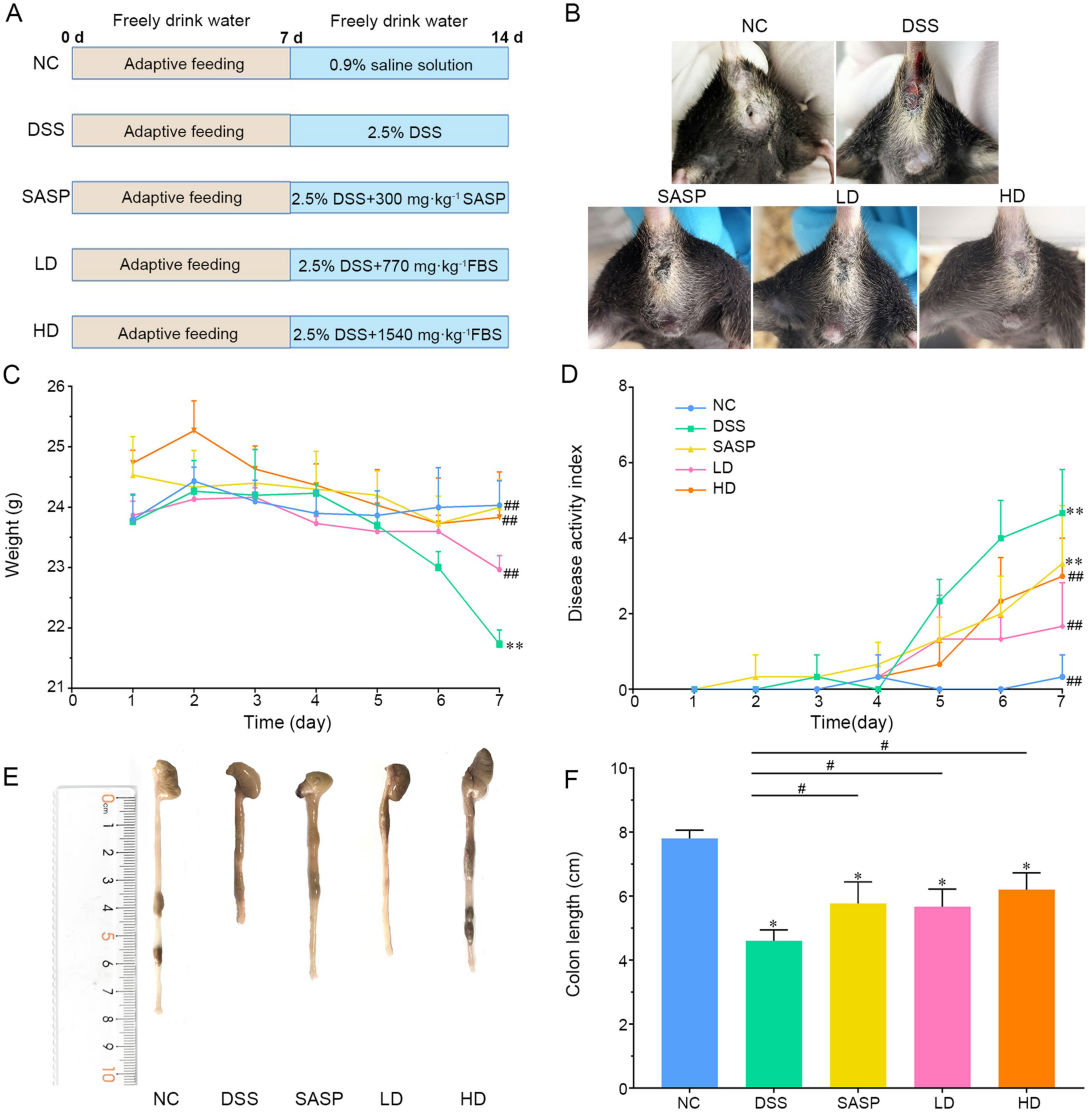
Fermented bamboo shoots alleviated clinical symptoms in UC mice. **(A)** Showed the schematic diagram of animal experiment. **(B)** Indicated the blood in the stool of mice. **(C)** Exhibited the changes of the body weight. **(D)** Presented the disease activity. **(E)** Meant the colon morphology in each group of mice. **(F)** Expressed the colon length in each group of mice. NC, control group; DSS, dextran sulfate sodium model group; SASP, drug positive group (Salazosulfapyridine); HD, high dose group; LD, low dose group; FBS, the fermented Bamboo shoot. Statistical analysis of the weight, the disease activity index (DAI) score and the colon length were performed using one-way ANOVA followed by Duncan’s multiple range test. Data are presented as mean ± SD. ^*^p < 0.05 and ^**^p < 0.01 versus NC group; *p* < 0.05 and ^##^*p* < 0.01 versus DSS group.

Sixty C57BL/6J male SPF mice, aged 6–8 weeks (weight 22 ± 2 g), were randomly divided into 5 groups after they had adapted to feeding for 7 days (12 rats in each group, *n* = 12). The experimental groups were as follows: normal control group (saline, NC), DSS model group (2.5% DSS, DSS), drug-positive group (2.5% DSS + 300 mg/kg salazosulfapyridine, SASP), low-dose group (2.5% DSS + 770 mg/kg fermented bamboo shoot, LD) and high-dose group (2.5% DSS + 1,540 mg/kg fermented bamboo shoot, HD) (Figure 1A). The basis for the dosage of the experimental groups above was the research of Liu et al. (22, 23), with small changes based on preliminary experiments. During the DSS induction period (8–14 days), daily body weight measurements were recorded, and the activity levels and anal conditions were observed. To assess disease severity in mice with ulcerative colitis, the disease activity index (DAI) was calculated using the scoring criteria proposed by Choi et al. (24). At the end of the experiment, the mice in each group were fasted for 12 h and sacrificed by decapitation. The colon was removed quickly, photographed, measured for length, and then divided into three segments. Two of these were stored at −80 °C and one was immersed in a 4% neutral formaldehyde solution. The caecum contents were also collected and stored at −80 °C. The experiment was approved by the ethical review committee for animal experiments of Guizhou Medical University (No. 2303225).

### 2.4 HE and AB-PAS staining

Colon samples were dehydrated, embedded and sectioned (4 μm thickness) in a 4% neutral formaldehyde solution. The sections were stained with haematoxylin and eosin (HE) and blocked to prepare them for examination by light microscopy. The above colon tissues, which were Paraffin-embedded, were stained with Alcian blue/periodic acid-Schiff (AB/PAS). The staining procedure was carried out in strict accordance with the kit manufacturer’s instructions. The images were viewed at 20x and 100x using a Nikon Eclipse Ts2R + FL microscope. The histopathological scoring criteria were based on the method described by Li et al. (25).

### 2.5 Determination of inflammatory factor levels

Pre-chilled phosphate-buffered saline containing protease inhibitor was mixed at a ratio of 100:1. Colon tissue was added at a weight-to-volume ratio of 1:9. Steel beads were then incorporated into the mixture and ground three times using a grinder at 60 Hz for 120 s per. Following this, the samples were removed and the mixture was then centrifuged at 4 °C (11,180 × *g* for 10 min). The supernatant was then extracted to measure the levels of TNF-α, IL-1β and IL-6 in the colon tissues of the mice according to the instructions of the ELISA kits.

### 2.6 Analysis of the gut microbiota

DNA from caecum contents was extracted using the hexadecyltrimethylammonium bromide (CTAB) method in accordance with the manufacturer’s instructions. DNA quality and purity were assessed by 2% agarose gel electrophoresis and NanoMicro-Drop-2000 spectrophotometer (Thermo Scientific, Wilmington, United States), respectively. The DNA gel recovery kit was used to retrieve the PCR products. The PCR primers 341F (5′-CCTACGGGNGGCWGCAG-3′) and 805R (5′-GACTACHVGGGTATCTAATCC-3′) were produced according to the V3–V4 section of the bacterial 16S rRNA gene, and the target pieces were copied by PCR. The PCR products were purified using AMPure XT beads (Beckman Coulter Genomics, Danvers, MA, United States), and quantitation was performed using a Qubit instrument (Invitrogen, United States). The purified PCR productions were then evaluated using an Agilent 2100 Bioanalyzer (Agilent, United States) and an Illumina quantification kit (Kapa Biosciences, Woburn, MA, United States), with qualified library concentrations set at above 2 nM. The Illumina NovaSeq PE250 platform was used to sequence the libraries at LC-Bio Technology Co., Ltd., in Hangzhou, Zhejiang Province, China. The NovaSeq 6000 sequencer from LC-Bio Technologies (Hangzhou) Co., Ltd. was used to perform the sequencing, with 2 × 250 bp paired-end reads created using the NovaSeq 6000 SP Reagent Kit (500 cycles). The raw sequence data were decoded using the Demux plugin and trimmed using the Cutadapt plugin. The DADA2 plugin was then used to process the sequences for quality filtering, denoising, assembly and removal of chimeras (26). On the basis of the obtained ASV sequences and abundance tables, the data were screened and analysed for alpha and beta diversity. Species annotation was based on ASV sequence files using the SILVA (NCBI repository, https://www.ncbi.nlm.nih.gov/, accession no. PRJNA1377287) database with NT-16S, and species abundance in each sample was based on the ASV (feature) abundance table. Microbiome analysis was based on 6 randomly selected mice (*n* = 6) from the 12 rats in each group.

### 2.7 Short-chain fatty acids

Short-chain fatty acids were isolated according to the methods of Inoue et al. (27) and Chen et al. (28), with modifications. Fifty milligrams of the sample were added to 1 mL of an 80% methanol solution. The mixture was ground and vortexed for 20 min, after which the extracted samples were placed at −20 °C for 30 min to precipitate the protein. Next, the samples were centrifuged at 19,999 *×* g for 15 min at 4 °C. Twenty microliters of the supernatant was derivatized with 20 μL of 1-(3-dimethylaminopropyl)-3-ethylcarbodiimide solution (96 mmol/L) and 20 μL of 3-nitrophenylhydrazine (200 mmol/L) in a 30 °C water bath for 1 h. The mixture was then diluted to 500 μL with methanol and acetonitrile at a 1:1 volume ratio, vortexed and mixed for UPLC-MS/MS analysis. UPLC was analysed using an Agilent Poroshell 120 EC-C18 column (2.1 mm × 100 mm, 2.7 μm). The solvent system used contained a mobile phase of water (A) and a mixture of methanol and acetonitrile at a ratio of 1:1 by volume (B). The gradient programme was as follows: phase A was set at 75.0% for 0.5 min, decreasing first to 45.0% at 13.0 min and then to 20.0% at 15.8 min. Phase A was then maintained at 75.0% for 18.0 min, before returning to 75.0% at 15.9 min.

An AB4500-QTRAP-UPLC/MS/MS system with integrated linear ion trap (LIT) technology and triple quadrupole (QQQ) mass spectrometry was used to analyse the sample extracts. The system was operated and controlled using Analyst 1.6.3 software (developed by ABSciex), which was equipped with an electrospray ionisation (ESI) turbo spray interface. The system had the following ESI source operating parameters: the ion source temperature was 400 °C; ion spray (IS) voltage was −3,000 V (negative mode); the ion source gas I (Gas1), gas II (Gas2) and curtain gas were set to 50, 60 and 25 psi, respectively. The collision-induced dissociation (CID) parameter was set to high intensity. The data acquisition modes included multiple reaction monitoring (MRM) and negative ion mode-specific analysis. Qualitative analysis of the target compounds was preformed using the retention time and MRM characterization of the fragment ions in the standard method. Quantitative analysis was then carried out using external standardisation by construction of a standard curve (Supplementary Table S1).

### 2.8 Statistical analysis

All the data were statistically analysed using IBM SPSS Statistics 22.0, and graphics were created using GraphPad Prism 8.0 and Photoshop 6.0. *p* < 0.05 was considered statistically significant. The columns and bars in identical graphs are expressed as the means ± standard deviations (SDs) of 6 biological replicates. One-way ANOVA with Duncan’s method was used for comparisons between groups. R version 4.1.3 (ggplot2 package version 3.4.0) was used to construct the Venn diagram. The stacked bar chart was created using R version 4.1.2 (ggplot2 package version 3.3.5). An advanced clustering heatmap was generated with the help of R version 4.2.0 (ComplexHeatmap package 2.12.0). The following bioinformatic analyses were performed using the OmicStudio tools at https://www.omicstudio.cn/tool: principal component analysis (PCA), principal coordinate analysis (PCoA), nonmetric multidimensional scaling analysis (NMDS), corrheatmap, relevance network diagram and false discovery rate (FDR) calculated with Benjamini–Hochberg method.

## 3 Results and discussion

### 3.1 Effects of fermented bamboo shoots on DSS-induced symptoms in mice

Body weight, DAI and colon length are important indicators for assessing UC symptoms in mice. During the experiment, the faeces of the DSS-treated mice gradually became soft and loose within 7 days of the administration of 2.5% DSS, and their colour changed from yellow to yellow-brown. Later, varying degrees of inflammation occurred, as indicated by blood in the stool, watery diarrhoea and anal bleeding (Figure 1B), suggesting that the gut microenvironment of the mice was gradually being damaged. In the mouse intervention experiment, the body weight of the mice in the model group decreased significantly from day 5 onwards, compared with those of the control group (*p* < 0.01). There was no significant difference in final body weight between the SASP, LD and HD groups (*p* > 0.05), but there was a significant difference compared the DSS group (*p* < 0.01) (Figure 1C). The increase in DAI in the DSS group was greater than that in the NC (*p* < 0.01) and LD groups (*p* < 0.05) (Figure 1D). Compared with that in the NC group, the colon length in the DSS group was significantly shorter (*p* < 0.05) and there was more pronounced bleeding and swelling. However, the SASP, LD and HD groups effectively alleviated colon shortening in the UC group (*p* < 0.05) (Figures 1E,F). The DSS group exhibited obvious symptoms of UC, such as weight loss, diarrhoea and blood in the stool. These symptoms are commonly used in DSS-induced UC models to evaluate pathogenesis and new therapeutic strategies (29). Measuring colon length further confirmed the degree of inflammation in colitis, and increasing DAIs confirmed the validity of the UC mouse model. Thus, changes in body weight, DAI and colon length are important in characterizing the severity of colitis in mice (30). These results suggest that the characteristics of DSS-induced colitis observed in the mice in this study are similar to those seen in previous studies (31, 32). The above results show that fermented bamboo shoots can significantly reduce the high DAI induced by DSS, effectively controlling shortened colon length. This indicates that fermented bamboo shoots can effectively relieve the symptoms of UC in mice.

### 3.2 Effects of fermented bamboo shoots on the colonic morphology of mice with colitis

**Figure 2:**
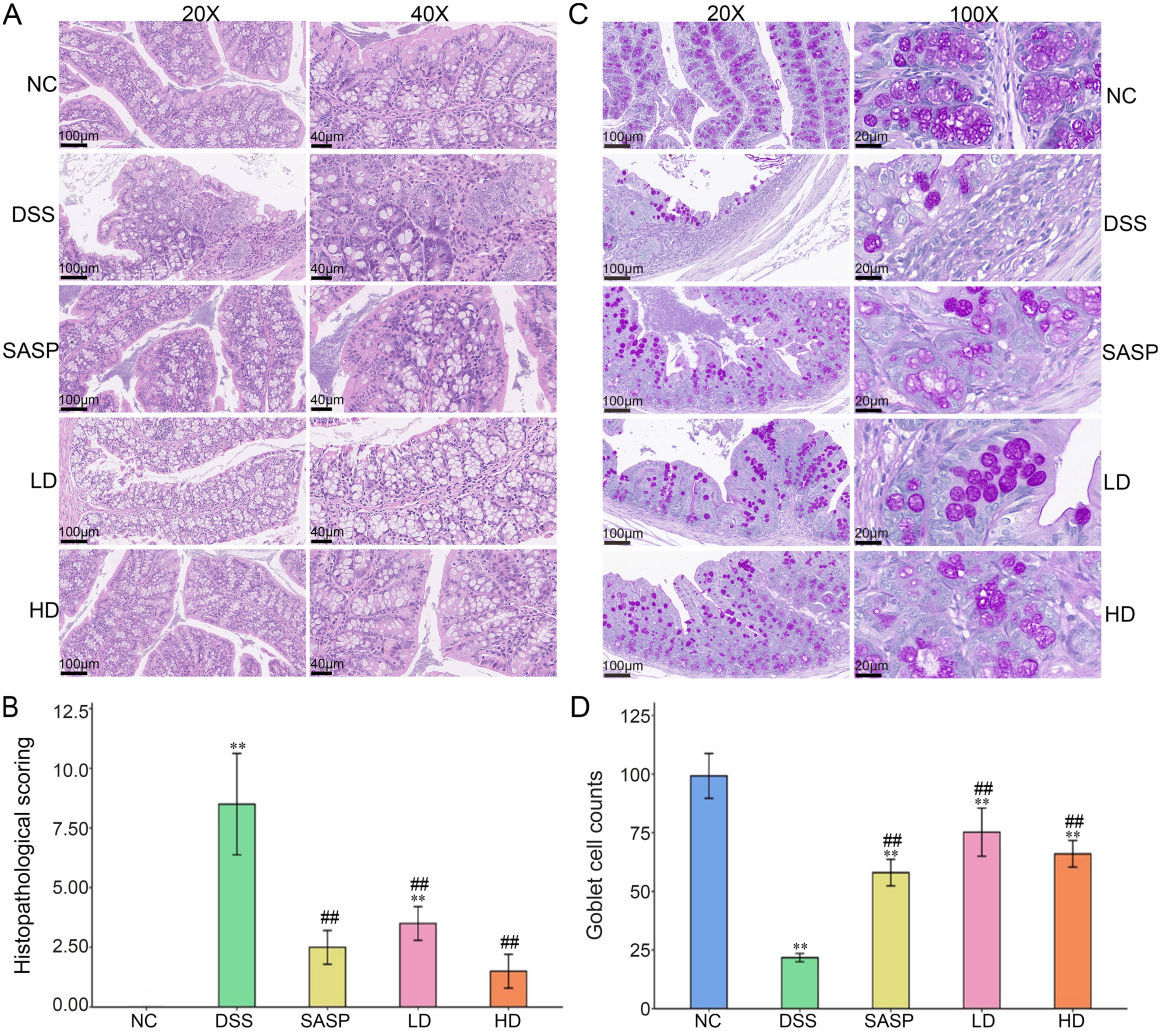
Histopathological staining and scoring of mouse colon tissue. **(A)** Showed the H&E staining. **(B)** Exhibited the semi-quantitative histopathological scoring. **(C)** Meant the AB/PAS staining. **(D)** Expressed the goblet cell counts. H&E, haematoxylin and eosin stain; AB/PAS, Alcian blue/periodic acid-Schiff; NC, control group; DSS, dextran sulfate sodium model group; SASP, drug positive group (Salazosulfapyridine); HD, high dose group; LD, low dose group. Statistical differences were determined by one-way ANOVA followed by Duncan’s multiple range test. Data are presented as mean ± SD. ^*^p < 0.05 and ^**^p < 0.01 versus NC group; *p* < 0.05 and ^##^*p* < 0.01 versus DSS group.

The existing literature indicated the reduction in goblet cell numbers and destruction to the crypts with the colonic mucous layer were important signals of disrupted epithelial function in mice colitis (33, 34). However, the proliferation and replacement of damaged epithelial cells were driven mainly by transit-expanding cells in the crypt (33). Therefore, restoring intestinal function in UC hinged on addressing these factors. As shown in Figure 2A, colon tissue histopathological staining revealed a loss of goblet cells and infiltration of inflammatory cells in the submucosa of the DSS group compared to NC group. Compared with the DSS and SASP groups, the intervention with fermented bamboo shoots in the LD and HD groups alleviated the area of colonic inflammation, the inflammatory cell infiltration was significantly improved, and the loss of epithelial cells was reduced. Additionally, the histopathological scores in the SASP, LD and HD group were significantly lower than those in the DSS group (*p* < 0.05) (Figure 2B). These findings suggest that DSS-induced disruption of the intestinal mucus layer in mice may lead to the inhibition of neoepithelial cell proliferation, whereas fermented bamboo shoots may improve the integrity of the colonic mucosa by promoting epithelial cell proliferation.

AB-PAS staining is commonly used to detect mucin in the colon. The lack of mucin, an important component of mucus layer production, may directly trigger UC (35). PAS staining revealed that the goblet cells in the NC group secreted normal levels of mucin, which was visible in the colon (Figure 2C). DSS induced cellular infiltration and a decrease in the number of goblet cells, which was consistent with the changes in DAI and colon morphology (Figures 1, 2A,D). Compared with the DSS group, the SASP, LD, and HD groups presented better protection, preserving the crypt structure, reducing goblet cell damage, and maintaining mucin secretion. In our study, the intervention involving fermented bamboo shoots alleviated the loss of goblet cells and the reduction in crypt depth in mice treated with DSS-treated. Notably, the intestinal mucosal conditions of the SASP group were similar to those of the LD and HD groups. Taken together, these findings suggest that fermented bamboo shoots may protect against UC by maintaining the integrity of the intestinal mucosal goblet cells and crypt structure.

### 3.3 Effects of fermented bamboo shoots on the levels of inflammatory cytokines induced by DSS in mice

**Figure 3:**
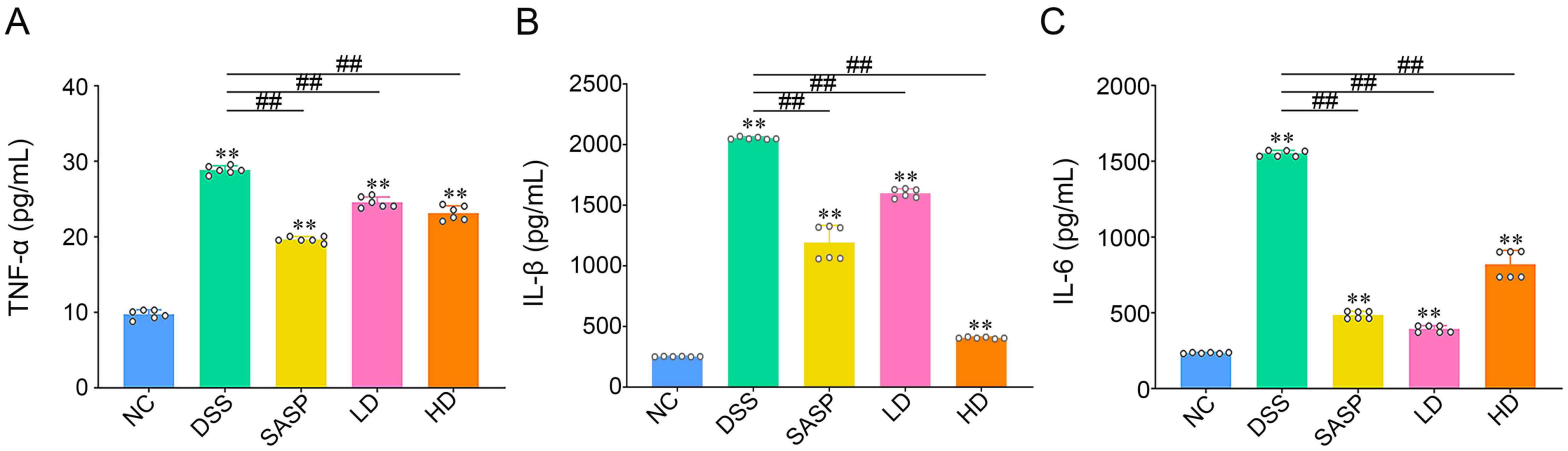
The expression levels of colon tissue cytokines in mice. **(A)** Meant the expression levels of TNF-α. **(B)** Showed the expression levels of IL-1β. **(C)** Exhibited the expression levels of IL-6. NC, control group; DSS, dextran sulfate sodium model group; SASP, drug positive group (Salazosulfapyridine); HD, high dose group; LD, low dose group. Statistical differences were determined by one-way ANOVA followed by Duncan’s multiple range test. Data are presented as mean ± SD. ^*^p < 0.05 and ^**^p < 0.01 versus NC group; *p* < 0.05 and ^##^*p* < 0.01 versus DSS group.

The levels of TNF-α, IL-6 and IL-1β in the colon tissues of the mice were detected to evaluate the anti-inflammatory effects of the fermented bamboo shoots. As shown in Figure 3, the levels of TNF-α, IL-6 and IL-1β in the DSS group were significantly greater than those in the NC, SASP, LD, and HD groups (*p* < 0.01), with the highest levels being 28.87 pg/mL, 2,053.43 pg./mL and 1,566.96 pg/mL, respectively. In the study by Shi et al. (36), elevated levels of TNF-α, IL-1β, and IL-6 were similarly detected in the serum of DSS-treated UC mice. These findings indicate that DSS induction aggravated inflammation in colon tissue. Although TNF-α, IL-6 and IL-1β levels were significantly greater in the LD and HD groups than in the NC group, the levels of these three inflammatory factors in the LD and HD groups were similar to those in the SASP group. Immune factors significantly affect intestinal health, and excessive inflammation can lead to colonic damage, such as increased levels of TNF-α, IL-6 and IL-1β in mice with colitis, which can significantly aggravate the symptoms of intestinal inflammatory disease (37). Previous studies have shown that the activation of innate immune cells in the gut (such as macrophages and neutrophils) can be triggered by DSS, resulting in a massive release of pro-inflammatory cytokines (such as TNF-α, IL-6 and IL-1β), and thereby inducing acute or chronic inflammatory responses locally in the intestine (38). These results suggest that fermented bamboo shoots intervention leads to the suppression of inflammatory cytokine levels in UC model mice, indicating that fermented bamboo shoots have a significant effect on relieving UC.

### 3.4 Effects of dietary fermented bamboo shoots on the gut microbiota diversity in UC model mice

**Figure 4:**
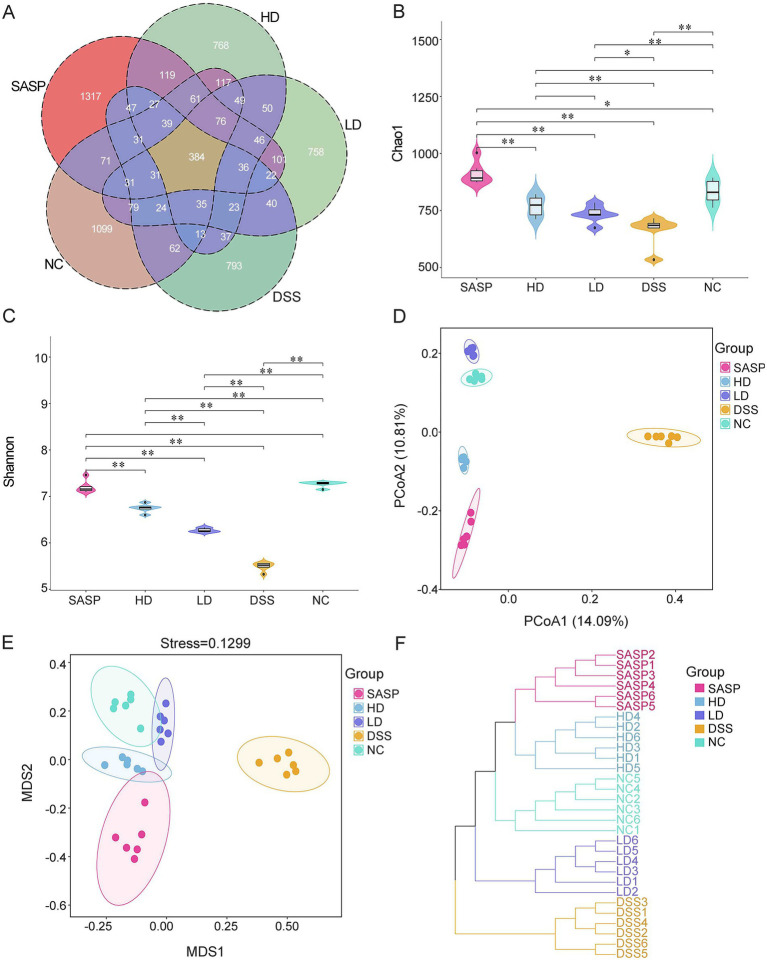
Effects of fermented bamboo shoots on gut microbiota diversity in mice. **(A)** Showed the Venn diagram. **(B)** Indicated the Chao1 index based α-diversity. **(C)** Meant Shannon index based on α-diversity. **(D)** Presented the principal coordinate analysis (PCoA) based on β-diversity. **(E)** Exhibited the non-metric multidimensional scaling analysis (NMDS) based on β-diversity. **(F)** Expressed the hierarchical cluster analysis of β-diversity for the gut microbiota in each group of mice. MDS, multidimensional scaling analysis; NC1/2/3/4/5/6, the repeat for control group; DSS1/2/3/4/5/6, the repeat for dextran sulfate sodium model group; SASP1/2/3/4/5/6, the repeat for drug positive group (Salazosulfapyridine); HD1/2/3/4/5/6, the repeat for high dose group; LD1/2/3/4/5/6, the repeat for low dose group.

The Venn diagram provides a visualization of the overlap and independence of the operational taxonomic units (OTUs) in the different treatment groups. Figure 4A illustrated the OTUs derived from the Venn diagram results, showing 2,202, 1,664, 2,439, 1,785 and 1,880 unique OTUs for the NC, DSS, SASP, LD, and HD groups, respectively. The total number of OTUs across all 6 treatment groups is 384. The number of OTUs in the LD and HD groups increased following fermented bamboo shoots intervention, compared with those in the DSS group. Improved after. These findings indicated that the intestinal microenvironment was altered to a greater extent by fermented bamboo shoots. Analysing the α-diversity of the gut microbiota is a key method for assessing the abundance and uniform distribution of taxa among the groups in samples. This study evaluated this using the Chao1 and Shannon indices in this study (Figures 4B,C). The Chao1 and Shannon indices were significantly lower in the DSS group than in the NC group, indicating that species distribution diversity and richness were greatly reduced in the DSS group. Similar results were found in the DSS group, compared with the SASP, LD and HD groups. Additionally, the Chao1 and Shannon indices were also significantly higher in the LD and HD groups than in the DSS groups. It is worth noting that the Chao 1 indices in the HD group were not significantly different to those in the LD or NC groups. β-diversity analysis uses PCoA and NMDS to calculate a distance matrix between samples, reflecting the similarities and differences between microbial communities within each group. As shown in Figures 4D,E, the results of PCoA and NMDS also revealed that the gut microbiota structure of the DSS group differed significantly from that of the NC, HD and LD groups. There was a tendency for the HD and LD groups to cluster with the NC group. Hierarchical cluster analysis (HCA) can be used to visually classify samples into different groups or clusters. Figure 4F shows that HCA classified a total of 30 samples from 5 groups into 4 classes: SASP and HD were classified together, and the remaining DSS, LD and NC as one class each. In terms of major group classification, DSS constitutes one category, with the remaining four groups forming another. These results indicate that intervention with fermented bamboo shoots may exert a regulatory effect on DSS-induced changes in gut microbiota structure.

### 3.5 Effects of fermented bamboo shoots on the gut microbiota structure in UC mice

The top 30 phyla of bacteria with the greatest changes in abundance in each group of mice are shown in Supplementary Figure S2A. Among these, the top 6 phyla in each group of mice were Firmicutes, Bacteroidota, Proteobacteria, Desulfobacterota, Verrucomicrobiota and Actinobacteriota. In addition, DSS induction resulted in abnormal changes to the composition of gut microorganisms, compared to the NC group. Specifically, there were significant increases in the relative abundance of the Firmicutes (10.07%), Actinobacteria (1.81%) and Patescibacteia (1.22%), and significant decreases in the relative abundance of Bacteroidota (8.31%) and Verrucomicrobiota (1.98%). This finding is consistent with the results of previous studies which reported an abnormal increase in the abundance of Actinobacteria and Patescibacteria, and a decrease in the abundance of Verrucomicrobiota, in the intestines of patients with UC (39, 40). Compared with those in the other groups, the abundance of Proteobacteria and Myxococcota was significantly higher in the LD group, while the abundance of Verrucomicrobiota was significantly higher in the HD group. Moreover, the Bacteroidota has been shown toto efficiently catabolise metabolised hydrocarbons to produce SCFAs, thereby alleviating UC (41). In our study, the LD group showed a significant increase in Bacteroidota abundance (4.81%) compared to the DSS group. These results suggest that fermented bamboo shoots can regulate the composition of gut microbiota at the phylum level by decreasing the relative abundance of Firmicutes and Actinobacteria, while increasing that of Bacteroidota.

**Figure 5:**
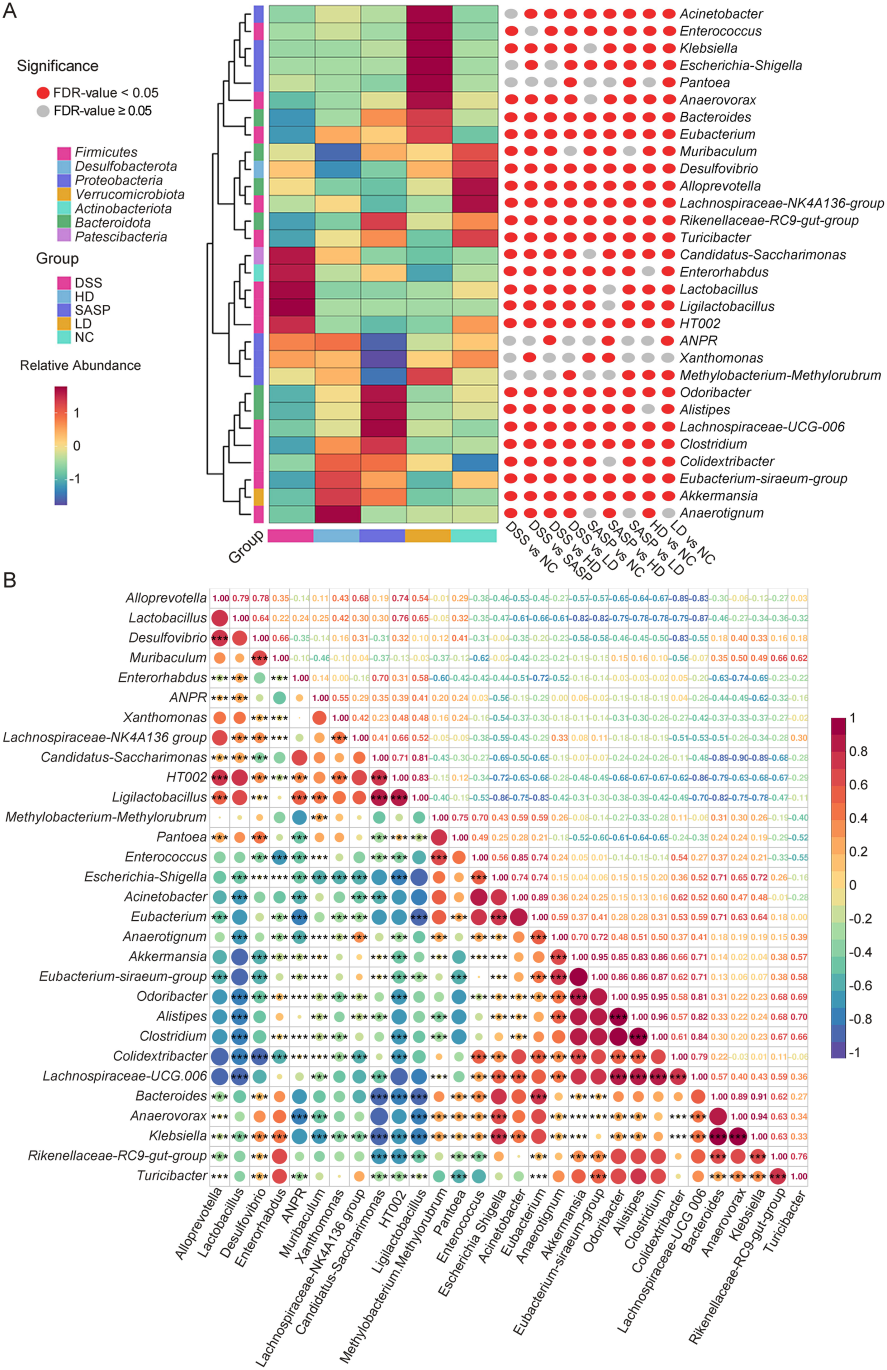
Significant difference and correlation analyses of gut microbiota in each group at the genus level with the top 30 abundance classification. **(A)** Indicated the advanced clustering heatmap. **(B)** Showed the correlation heatmap. FDR, false discovery rate; NC, control group; DSS, dextran sulfate sodium model group; SASP, drug positive group (Salazosulfapyridine); HD, high dose group; LD, low dose group; ANPR, *Allorhizobium-Neorhizobium-Pararhizobium-Rhizobium*. The colour scale indicates the correlation coefficient. Statistical differences were determined by one-way ANOVA followed by Duncan’s multiple range test. ^*^p < 0.05, ^**^p < 0.01, and ^***^p < 0.001.

The top 30 genera with the greatest changes in abundance are shown in Figure 5A; Supplementary Figure S2B. Compared with the NC group, the DSS group presented a significant increase in the relative abundance of 4 genera (significantly different screening criteria of both a fold change >2 and FDR <0.05), including *Ligilactobacillus*, *Lactobacillus*, *Enterorhabdus*, and *Candidatus_Saccharimonas* In addition, 13 genera in the DSS group, including *Lachnospiraceae_NK4A136_group*, *Alloprevotella*, *Clostridium*, *Bacteroides*, *Akkermansia*, *Alistipes*, *Eubacterium*, *Eubacterium_siraeum_group*, *Turicibacter*, *Odoribacter*, *Rikenellaceae_RC9_gut_group*, *Klebsiella*, *Anaerovorax* and *Enterococcus*, significantly decreased in relative abundance compared with those in the NC group. Among these 15 genera, 4 genera (*Ligilactobacillus*, *Lactobacillus*, *Enterorhabdus* and *Candidatus_Saccharimonas*) presented significant decreases in abundance, and 11 genera (*Clostridium*, *Bacteroides*, *Akkermansia*, *Alistipes*, *Eubacterium*, *Eubacterium_siraeum_group*, *Turicibacter*, *Odoribacter*, *Rikenellaceae_RC9_gut_group*, *Klebsiella* and *Anaerovorax*) presented significant increases in relative abundance after fermented bamboo shoots intervention. A previous study revealed that the enrichment of *Candidatus_Saccharimonas* could trigger inflammatory effects in the intestinal mucosa (42). When considered alongside the results shown in Figures 3, 5A, these findings further explain why the DSS-induced model groups all presented higher levels of inflammatory factors than did the other groups. In addition, *Akkermansia* belongs to the Verrucomicrobia phyla (Supplementary Figure S3). This phylum is comprised of probiotics, which play a vital role in maintaining barrier function, host metabolism and the immune response (43). *Alistipes* has anti-inflammatory properties and promotes the production of SCFAs, making it a probiotic that can help to reduce gut inflammation (44). These results suggest that fermented bamboo shoots can alter the abundance of gut microbiota at the genus level in UC model mice, thereby improving their intestinal microecological health.

Species with significant differences in abundance between groups were identified using the square root result of indicator (SqrtIVt) values and LDA effect size (LEfSe) (Supplementary Figures S4, S5). Compared to other groups, the DSS group exhibited significantly higher levels of the following genus: *Ligilactobacillus*, *Lactobacillus*, *Candidatus Saccharimonas* and *Enterorhabdus*. This indicated that these were the main characteristic bacterial group. Following the intervention with fermented bamboo shoots, the abundance levels of *Akkermansia*, *Eubacterium siraeum*, *Escherichia Shigella*, *Acinetobacter*, *Klebsiella* and *Enterococcus* also showed a significant increase. It was worth noting that *Akkermansia* can reduce the level of inflammation in the intestines and lessen the severity of colitis (43). Correlation analyses were performed on the top 30 gut microbiota in terms of relative abundance to explore the interactions between gut microbes (Figure 5B). Of them, the abundance of 6 bacterial species (including *Escherichia Shigella*, *Odoribacter*, *Alistipes*, *Clostridium*, *Turicibacter* and *Colidextribacter*) were positively correlated with that of *Akkermansia*, as indicated by a Pearson’s coefficient greater than 0.50. The above results, combined with the characteristic bacterial groups shown in Supplementary Figures S4, S5, suggest that fermented bamboo shoots effectively restore the gut microbiota structure of UC mice to that of normal mice.

### 3.6 Effects of fermented bamboo shoots on the short-chain fatty acid content of DSS-treated mice

**Figure 6:**
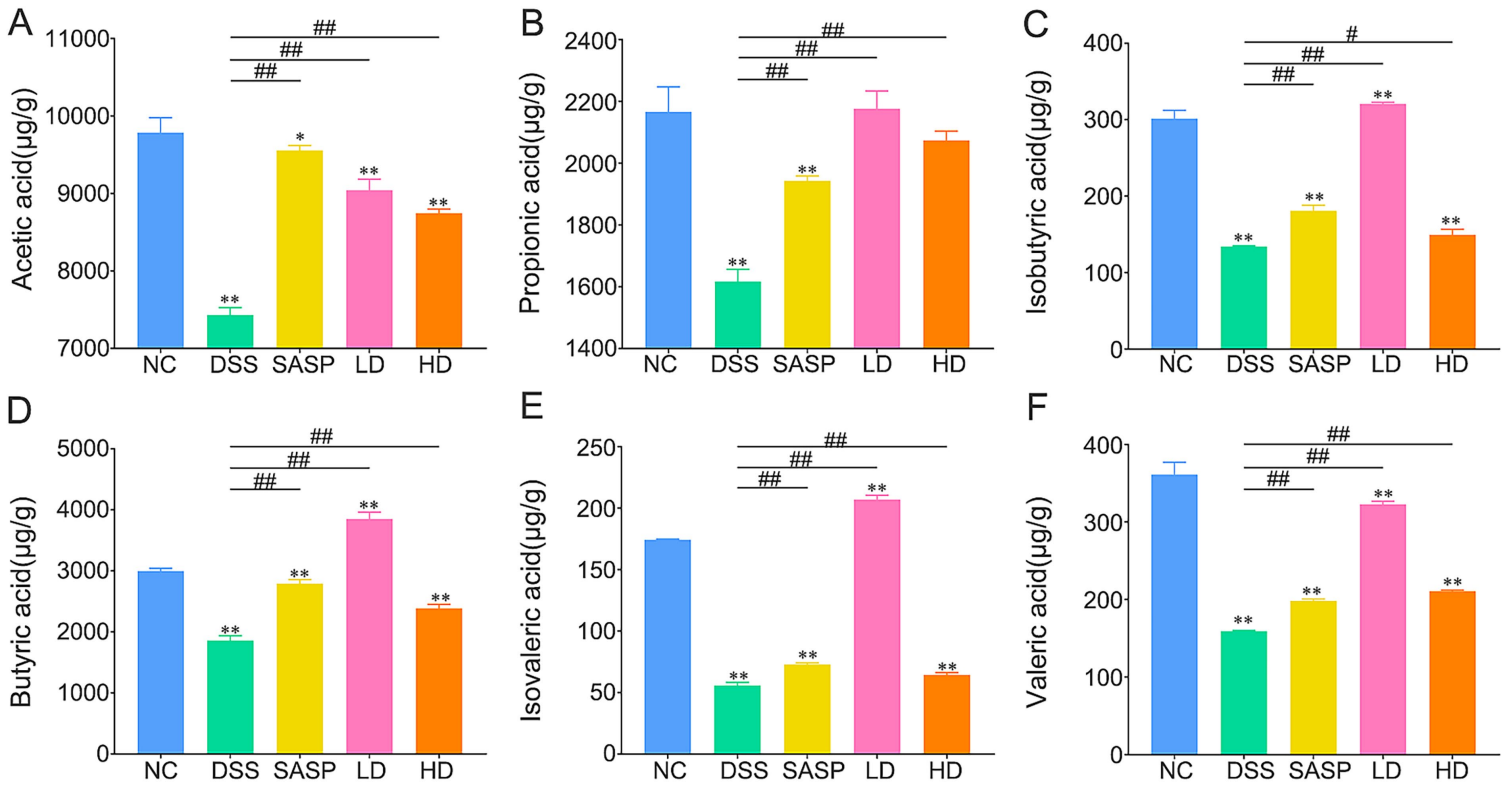
Effects of dietary fermented bamboo shoots on the short chain fatty acid in UC mice. **(A)** Showed the acetic acid content. **(B)** Indicated the propionic acid content. **(C)** Meant the isobutyric acid content. **(D)** Presented the butyric acid content. **(E)** Expressed the isovaleric acid content. **(F)** Exhibited the valeric acid content. NC, control group; DSS, dextran sulfate sodium model group; SASP, drug positive group (Salazosulfapyridine); HD, high dose group; LD, low dose group. Statistical differences were determined by one-way ANOVA followed by Duncan’s multiple range test. Data are presented as mean ± SD. ^*^p < 0.05 and ^**^p < 0.01 versus NC group; *p* < 0.05 and ^##^*p* < 0.01 versus DSS group.

SCFAs are important beneficial metabolites of the gut microbiota. They play a key role in maintaining host health by providing energy to the colonic epithelium, strengthening the intestinal barrier, inhibiting microbial pathogens and exhibiting anti-inflammatory properties (45). This study did not detect hexanoic acid in any of the groups. The qualitative methods and calibration equations for authentic standards of short-chain fatty acids are shown in Supplementary Table S1. As shown in Figures 6A–F, the levels of acetic, propionic, butyric, valeric, isobutyric and isovaleric acids were significantly lower in the DSS group than in the NC and SASP groups. However, all six short-chain fatty acids were significantly upregulated in the caecum content of UC mice after LD and HD interventions. In particular, the concentrations of isobutyric, butyric and isovaleric acid were higher in the LD group than in the control group. These results may also be related to the fact that the dominant bacterium in the LD group (Supplementary Figures S4, S5), is *Enterococcus*, which belongs to Firmicutes (Supplementary Figure S3), and butyrate is mainly produced by Firmicutes (46). *Enterococcus* can strengthen the intestinal mucosa and play a positive role in alleviating inflammation by producing short-chain fatty acids (47). Figure 6B shows that the proportionate level (2,176.67 μg/g) in the HD group were similar to the control level. Previous gut microbiota analysis revealed that the HD group had a gut microbiota dominated by *Akkermansia*, which was associated with the production of SCFAs, specifically, *Akkermansia muciniphila* produces propionate and acetate from mucin fermentation (48). *Akkermansia* relies on short-chain fatty acids to maintain immune balance while ensuring mucin secretion, increasing mucus thickness, and preserving the integrity of the intestinal mucosa (49). The above results suggest that supplementation with fermented bamboo shoots may promote the production of SCFAs and that these metabolites may help alleviate, to a certain extent, inflammation of the intestine and damage to the intestinal mucosa.

### 3.7 Correlation analysis of DSS-induced alterations in symptoms, inflammatory cytokines, short-chain fatty acids and the gut microbiota

**Figure 7:**
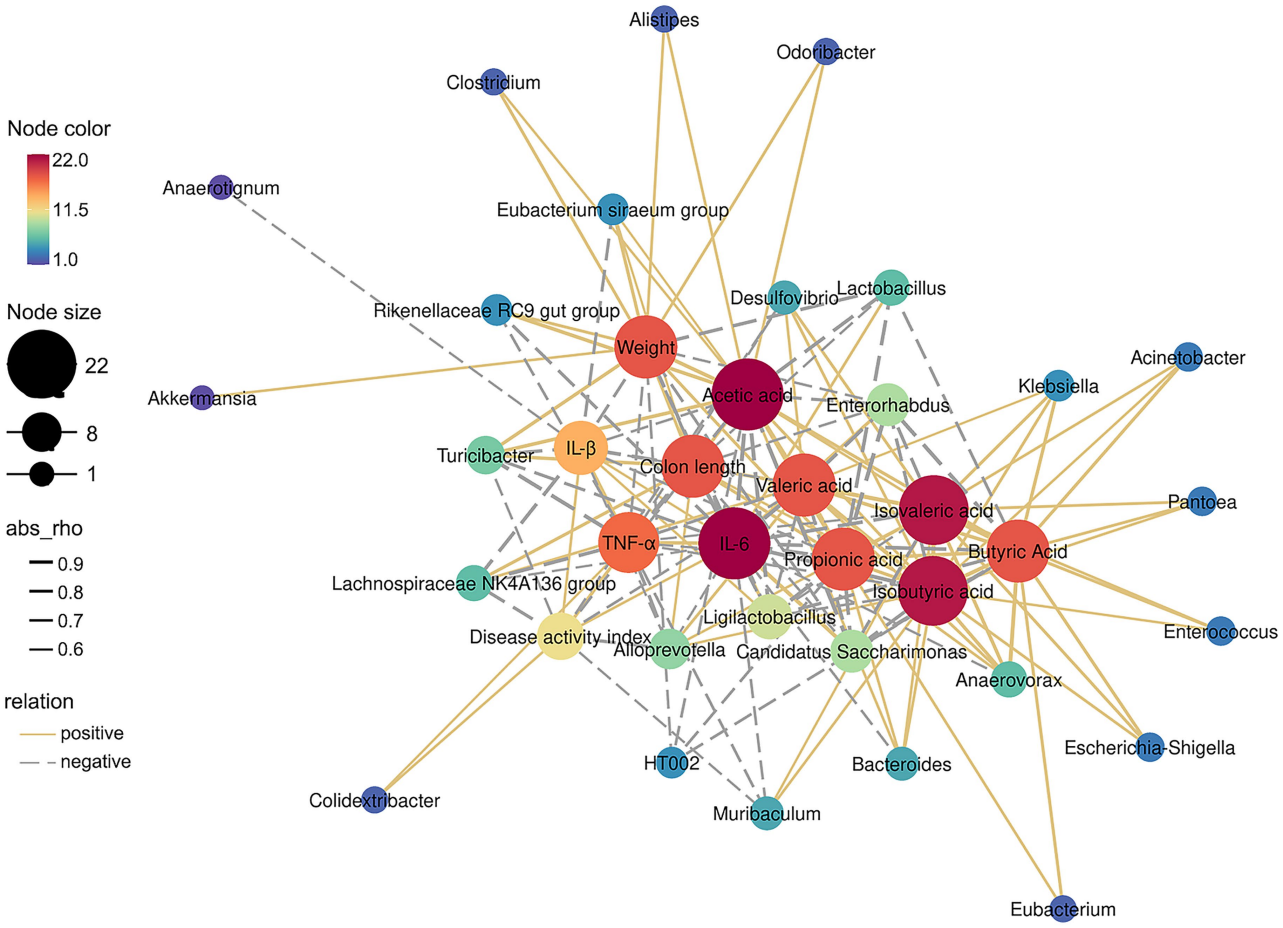
Relevance network diagram based on basic physicochemical indicators, inflammatory factors, short-chain fatty acids and gut microbiota. The microbiota at the genus level of the top 30 abundance classifications were the gut microbiota used for the relevance network diagram. The thickness of the connecting lines or abs_rho expressed the correlation coefficient, solid line indicated positive, dotted line indicated negative. Node colour or node size represented the number of relevant objects.

This study analysed the following by means of the Pearson coefficient: body weight, DAI score, colon length, colon inflammatory factors, the gut microbiota (the 30 most abundant bacteria species at the genus level) and the intestinal metabolite SCFAs were analysed by Figure 7. The genera *Candidatus_Saccharimonas*, *Enterorhabdus*, *Lactobacillus* and *Ligilactobacillus* were significantly enriched in the DSS group. They were positively correlated with the DAI score and 3 inflammatory cytokines, but negatively correlated with weight, colon length and 6 SCFAs. Qu D et al. also demonstrated that *Enterococcus faecalis* regulates cytokine production, thereby further suppressing colonic inflammation. This finding is consistent with the above findings (50). In addition, the modulation of inflammatory cytokine and SCFA levels is commonly associated with probiotics. It is possible that in some UC patients who have a weakened immune system, the beneficial bacteria *Lactobacillus* can also increase the risk of infection (51). Additionally, the abundance of *Turicibacter* was negatively correlated with the DAI and the levels of 3 inflammatory cytokines and positively correlated with body weight, colon length and the level of acetic acid. Among the dominant bacteria after the intervention of fermented bamboo shoots, the abundance of *Akkermansia* showed a strong positive correlation. *Anaerovorax* showed a strong positive correlation with 6 SCFAs and colon length, while strong negative correlations were observed with DAI, TNF-α and IL-6. Additionally, *Enterococcus* showed strong positive correlations with butyric acid, isovaleric acid, and isobutyric acid. Moreover, SCFAs, especially butyric acid and propionic acid, can inhibit the production of proinflammatory mediators (i.e., TNF-α, IL-6 and IL-β) through macrophages and neutrophils (52, 53). This observation could also explain why the above bacteria presented different correlations with inflammatory cytokine and SCFA levels. Taken together, these data suggest that changes in the abundance of characteristic gut microorganisms, such as *Akkermansia*, *Anaerovorax* and *Enterococcus*, are strongly associated with colitis severity and the production of inflammatory cytokines and SCFAs.

Previous study revealed that consuming organic acids produced during fermentation helps regulate gut microbiota and reduce the risk of chronic intestinal diseases (54). The accumulation or newly generated organic acids from fermented bamboo shoots provide crucial evidence for explaining observed changes in microbial community structure. These fermentation products alter the intestinal environment, creating a microenvironment conducive to the proliferation of SCFA-producing bacteria such as *Bacteroides* and *Verrucomicrobium* (55, 56). The abundance of amino acids may promote the colonization and growth of beneficial symbiotic bacteria such as *Akkermansia* (57). Similarly, differences in microbial community structure led to variations in the levels of their metabolic products, short-chain fatty acids. This suggests it may indirectly influence the host through the “microbiota-metabolite-immune axis.”

In addition, this study observed a dose–response relationship worthy of further investigation. For certain core indicators (such as body weight, disease activity index, inflammatory cell levels, and short-chain fatty acid content), the low-dose group showed a trend of superiority over the high-dose group. This phenomenon is not unique in natural product research, as demonstrated in Murakami (58), where low and moderate doses of plant polyphenols proved more beneficial for mice with colitis. Furthermore, considering the profound impact of fermented bamboo shoot products on the gut microbiota, high doses may disrupt the optimal balance of the intestinal microecology, thereby limiting the realization of their benefits. This discovery holds significant implications for human dietary practices. It suggests that moderate daily consumption of such products may serve as an effective strategy for maintaining gut health and preventing inflammatory diseases. Future research will focus on elucidating the underlying molecular mechanisms.

## 4 Conclusion

Studies have shown that the induction of DSS results in pathological abnormalities in mice, including weight loss, shortened colon length, elevated DAIs and proinflammatory cytokine levels, and disturbances in the intestinal flora. Fermented bamboo shoots ameliorate UC symptoms by decreasing proinflammatory cytokine levels (TNF-α, IL-1β and IL-6); increasing the abundance of SCFA-producing *Bacteroides*, *Verrucomicrobiota*, and *Akkermansia* species; and decreasing the abundance of *Enterorhabdus* and *Candidatus_Saccharimonas* abundance to modulate colon inflammation and restore intestinal microbiota homeostasis to some extent. Furthermore, there was a significant correlation between the abundance of gut microbes and the severity of colitis, inflammatory factor levels and SCFA levels, which interact in colitis model mice. This study highlights the potential application value of fermented bamboo shoots to alleviate intestinal inflammation and restore gut microbiota homeostasis in UC and provides a theoretical reference for research on the application of fermented bamboo shoots in the development of functional foods.

## Data Availability

The original contributions presented in the study are included in the article/Supplementary material, further inquiries can be directed to the corresponding authors.

## Supplementary material

SUPPLEMENTARY FIGURE S1 Component analysis of fermented and unfermented bamboo shoot products. **(A)** Meant differential metabolite analysis of fermented and unfermented bamboo shoot products. **(B)** Main constituents of fermented bamboo shoots. CK-1/2/3, the repeat for unfermented bamboo shoot products group. Treat-48h-1/2/3, the repeat for fermented bamboo shoot products group. RP1/2/3, the repeats for fermented bamboo shoots. Amino acids, amino acids and derivatives; nucleotides, nucleotides and derivatives.

SUPPLEMENTARY FIGURE S2 Stacked bar chart based on the gut microbiota selecting the abundance of the top 30 classified species in each group of mice. **(A)** Meant the stacked bar chart based on the phylum level. **(B)** Expressed the stacked bar chart based on the genus level. NC, control group; DSS, dextran sulfate sodium model group; SASP, drug positive group (Salazosulfapyridine); HD, high dose group; LD, low dose group; ANPR, *Allorhizobium-Neorhizobium-Pararhizobium-Rhizobium*.

SUPPLEMENTARY FIGURE S3 Sankey plot at the level of the phylum and genus for groups of the gut microbiota. NC, control group; DSS, dextran sulfate sodium model group; SASP, drug positive group (Salazosulfapyridine); HD, high dose group; LD, low dose group; ANPR, *Allorhizobium-Neorhizobium-Pararhizobium-Rhizobium*.

SUPPLEMENTARY FIGURE S4 Indicator analysis of gut microbiota in each group based on the abundance top 30 species. sqrtIVt, the square root result of indicator value. (A) meant the indicator analysis of gut microbiota based on the phylum level; (B) expressed the indicator analysis of gut microbiota based on based on the genus level. ANPR, *Allorhizobium-Neorhizobium-Pararhizobium-Rhizobium*; NC, control group; DSS, dextran sulfate sodium model group; SASP, drug positive group (Salazosulfapyridine); HD, high dose group; LD, low dose group.

SUPPLEMENTARY FIGURE S5 LEfSe analysis of the gut microbiota between the groups. LEfSe, linear discriminant analysis effect size (LDA effect size); NC, control group; DSS, dextran sulfate sodium model group; SASP, drug positive group (Salazosulfapyridine); HD, high dose group; LD, low dose group. The screening criteria used to identify species with a significant difference in abundance were both LDA values ≥ 3 and *p* < 0.05.
