# Supplementary figures and images for: Bamboo shoot fermented products alleviated DSS-induced ulcerative colitis in mice by effectively controlling inflammatory reactions and adjusting the gut microbiota and its metabolites
Source: Front Nutr. 2025 Dec 19;12:1724148. doi: 10.3389/fnut.2025.1724148 (PMC12757230; doi:10.3389/fnut.2025.1724148)

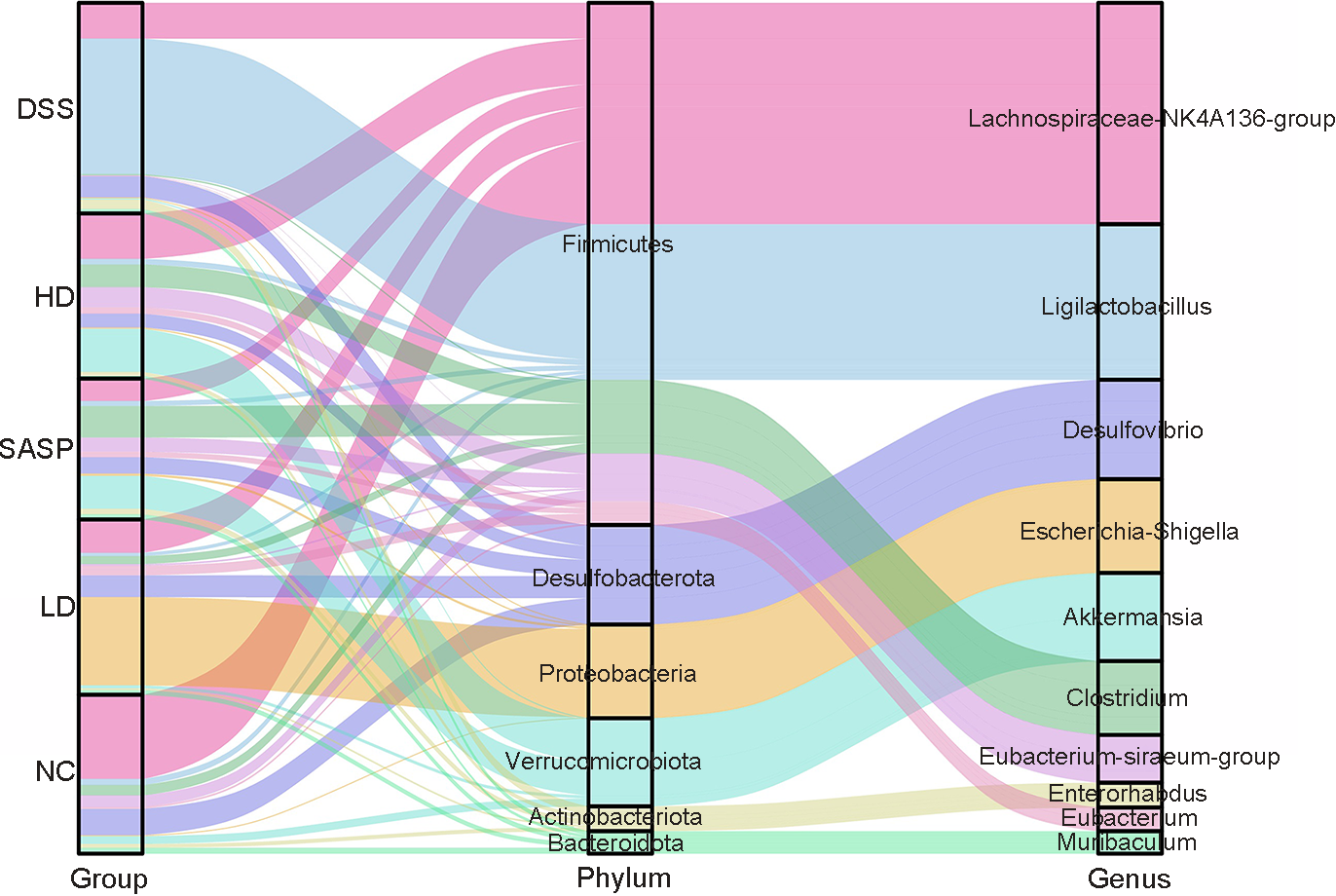

Supplement: SUPPLEMENTARY FIGURE S3 — Sankey plot at the level of the phylum and genus for groups of the gut microbiota. NC, control group; DSS, dextran sulfate sodium model group; SASP, drug positive group (Salazosulfapyridine); HD, high dose group; LD, low dose group; ANPR, Allorhizobium-Neorhizobium-Pararhizobium-Rhizobium. [file Image_3.TIF]

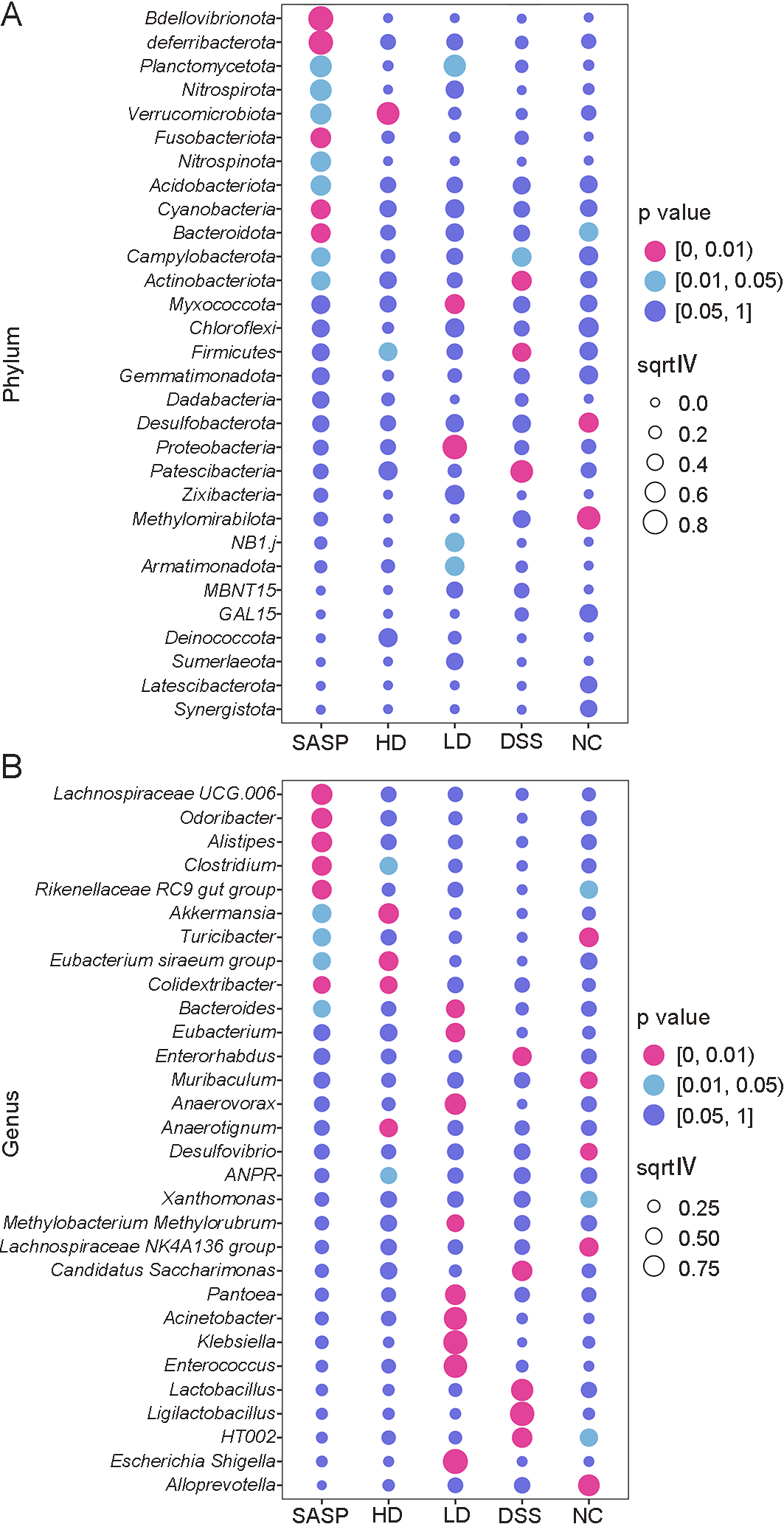

Supplement: SUPPLEMENTARY FIGURE S4 — Indicator analysis of gut microbiota in each group based on the abundance top 30 species. sqrtIVt, the square root result of indicator value. (A) meant the indicator analysis of gut microbiota based on the phylum level; (B) expressed the indicator analysis of gut microbiota based on based on the genus level. ANPR, Allorhizobium-Neorhizobium-Pararhizobium-Rhizobium; NC, control group; DSS, dextran sulfate sodium model group; SASP, drug positive group (Salazosulfapyridine); HD, high dose group; LD, low dose group. [file Image_4.TIF]

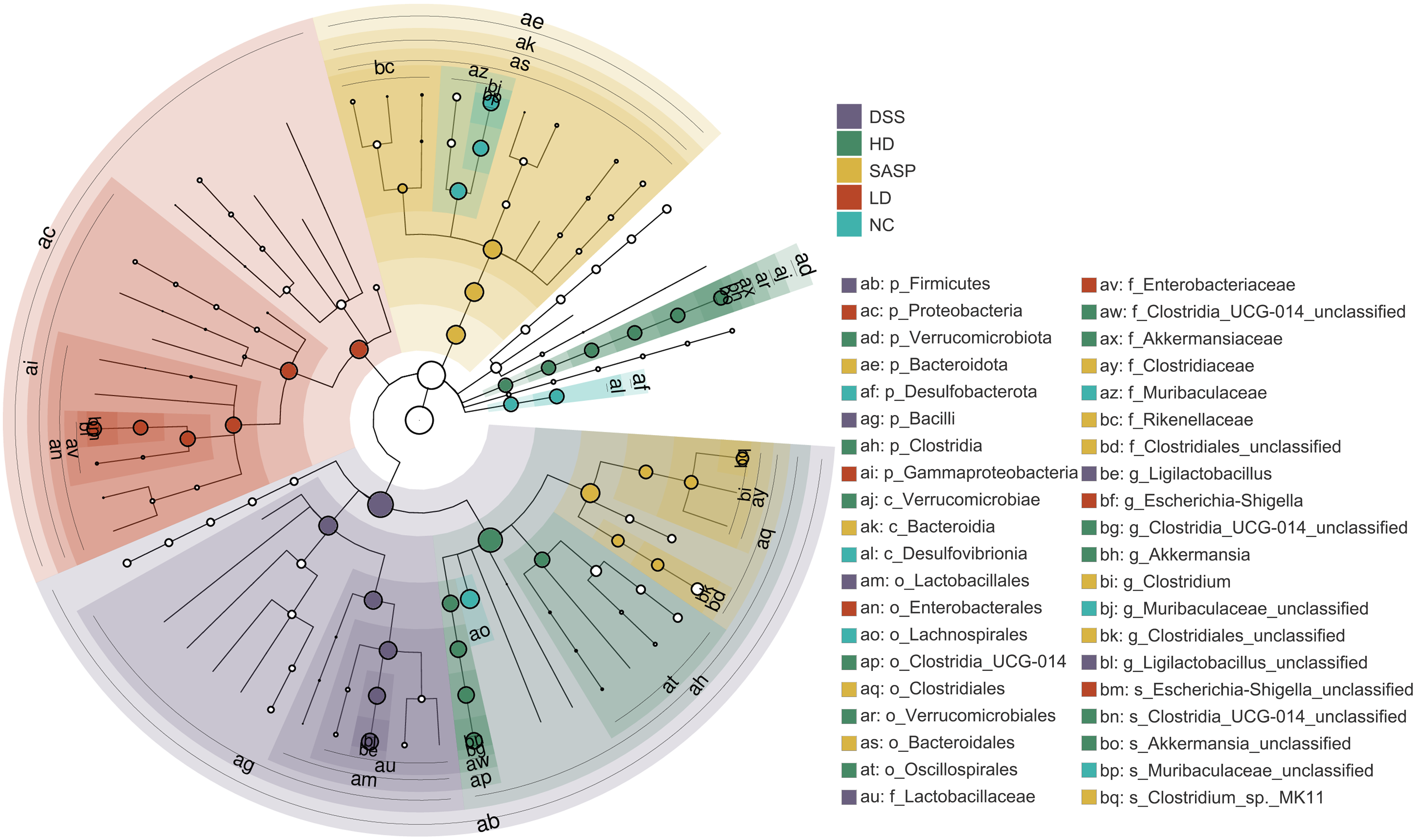

Supplement: SUPPLEMENTARY FIGURE S5 — LEfSe analysis of the gut microbiota between the groups. LEfSe, linear discriminant analysis effect size (LDA effect size); NC, control group; DSS, dextran sulfate sodium model group; SASP, drug positive group (Salazosulfapyridine); HD, high dose group; LD, low dose group. The screening criteria used to identify species with a significant difference in abundance were both LDA values ≥ 3 and p < 0.05. [file Image_5.TIF]
